# Vascular Reactions of the Diving Reflex in Men and Women Carrying Different ADRA1A Genotypes

**DOI:** 10.3390/ijms23169433

**Published:** 2022-08-21

**Authors:** Tatyana Baranova, Ekaterina Podyacheva, Tatyana Zemlyanukhina, Dmitrii Berlov, Maria Danilova, Oleg Glotov, Andrey Glotov

**Affiliations:** 1 Faculty of Biology, Saint Petersburg State University, 199034 Saint-Petersburg, Russia; 2 Faculty of Biology, Herzen State Pedagogical University of Russia, 191186 Saint-Petersburg, Russia; 3 Department of Genomic Medicine, D.O. Ott’s Institute of Obstetrics, Gynecology and Reproductology, 199034 Saint-Petersburg, Russia; 4 Department of Experimental Medical Virology, Molecular Genetics and Biobanking of Pediatric Research and Clinical Center for Infectious Diseases, 197022 Saint-Petersburg, Russia

**Keywords:** diving reflex, polymorphism *Arg347 ADRA1A (rs1048101)*, sexual dimorphism in human, pulmonary blood flow, pulmonary vascular tone, vasoconstriction, vasodilation

## Abstract

The diving reflex is an oxygen-saving mechanism which is accompanied by apnea, reflex bradycardia development, peripheral vasoconstriction, spleen erythrocyte release, and selective redistribution of blood flow to the organs most vulnerable to lack of oxygen, such as the brain, heart, and lungs. However, this is a poorly studied form of hypoxia, with a knowledge gap on physiological and biochemical adaptation mechanisms. The reflective sympathetic constriction of the resistive vessels is realized via ADRA1A. It has been shown that ADRA1A SNP (*p.Arg347Cys; rs1048101*) is associated with changes in tonus in vessel walls. Moreover, the Cys347 allele has been shown to regulate systolic blood pressure. The aim of this work was to evaluate whether the ADRA1A polymorphism affected the pulmonary vascular reactions in men and women in response to the diving reflex. Men (*n* = 52) and women (*n* = 50) untrained in diving aged 18 to 25 were recruited into the study. The vascular reactions and blood flow were examined by integrated rheography and rheography of the pulmonary artery. Peripheral blood circulation was registered by plethysmography. The ADRA1A gene polymorphism (*p.Arg347Cys; rs1048101*) was determined by PCR-RFLP. In both men and women, reflective pulmonary vasodilation did occur in response to the diving reflex, but in women this vasodilation was more pronounced and was accompanied by a higher filling of the lungs with blood.. Additionally, ADRA1A SNP (*p.Arg347Cys; rs1048101*) is associated with sex. Interestingly, women with the Arg347 allele demonstrated the highest vasodilation of the lung vessels. Therefore, our data may help to indicate women with the most prominent adaptive reactions to the diving reflex. Our data also indicate that women and men with the Cys allele of the *ADRA1A* gene polymorphism have the highest risk of developing lung hypertension in response to the diving reflex. The diving reflex is an oxygen-saving mechanism which is accompanied by apnea, reflex bradycardia development, peripheral vasoconstriction, spleen erythrocyte release, and selective redistribution of blood flow to the organs most vulnerable to lack of oxygen, such as the brain, heart, and lungs. However, this is a poorly studied form of hypoxia, with a knowledge gap on physiological and biochemical adaptation mechanisms.

## 1. Introduction

Our knowledge of the natural protective mechanisms against extreme environmental factors is the key to controlling the human body. Diving hypoxia is one of these extreme factors with poorly studied physiological and biochemical adaptation mechanisms. Currently, the diving reflex phenomenon has been described in detail [1] and is associated with cardiovascular reactions aimed at the effective use of oxygen during submersion [2]. The diving reflex also includes peripheral vessel constriction, selective redistribution of blood flow to the brain and heart, reflex bradycardia development [3,4,5,6], and spleen erythrocyte release [7]. In addition to this complex of reactions, blood pressure raising and significant changes in the pulmonary blood flow occur [8,9,10,11,12,13]. To our current knowledge, the slowing of blood flow due to a decrease in heart rate reduces oxygen consumption by the body. Interestingly, some literature data [14] and our studies [15] indicate an inverse correlation between the characteristics of reflex bradycardia and apnea duration during diving.

Currently, the fact of increased blood flow into the lungs during imitation of the diving reflex (face submersion into cold water) is well established [9]. This occurs due to a decreased air volume in the lungs and, as a consequence, an increased hyperbaric factor that affects the body during submersion. It is assumed that increased blood flow into the lung vessels has a protective effect and helps to maximize the chest volume [16]. Using the method of bioimpedance plethysmography, we recently reported pulmonary vessel dilation during imitation of the diving reflex with a latent period of 2–4 s, which indicated that the autonomic nervous system (ANS) acted prior to the hyperbaric factor [17]. It is known that the pulmonary vessels are innervated by the sympathetic and parasympathetic nerve fibers of the ANS. Most of the pulmonary sympathetic nerves are adrenergic and require α- and β-adrenergic receptors (ARs), which are expressed by the pulmonary endothelium and smooth muscle cells of the pulmonary vessels. Interestingly, the stimulation of the α_1_- and α_2_-ARs (with the predominant role of α_1A_-subtype receptors) in the pulmonary artery smooth muscle cells results in vasoconstriction [18,19,20]. In contrast, the β_1_- and β_2_-ARs in the vascular endothelium dilate the pulmonary vessels [21]. Our previous studies have shown that vasoconstriction of the peripheral vessels modulates the β_2_-ARs and bradykinin receptor B2 (BDKR B2), mediating dilation [22]. Moreover, we have demonstrated a high inter-individual variability of the peripheral vessels’ [23,24,25] and pulmonary vessels’ [17] constriction ability. This observation can be explained by at least two factors: (1) the genetic variation of the receptors that mediate vasoconstriction and vasodilation, and (2) the current hormonal status of a body (i.e., sex hormones).

Indeed, the α_1A_-ARs are a hub that controls the adrenergic reflective constriction of the resistive vessels. The adrenoceptor alpha 1A (*ADRA1A*) gene is localized in the 8p21.2 chromosome and has nine single-nucleotide polymorphisms (SNPs), where seven SNPs are associated with changes in the amino acid sequence of the α_1A_ protein. In the *ADRA1A* gene, the SNP rs1048101 (1039T > C) is one of the most frequent SNPs and is responsible for an *Arg347Cys (rs1048101)* substitution at the C-terminal of the α_1A_-ARs [26]. In European Americans, the allele frequencies are presented as 45% Arg and 55% Cys [27]. The Arg347Cys substitution can confer an additional palmitoylation site and may modulate C-terminal receptor conformational changes and its interaction with other proteins, with no significant influence on function in vitro [26]. However, in vivo studies demonstrated that genetic variations of this mutation may alter the sympathetic nervous system activity and contribute to the pathogenesis of hypertonia and cardiovascular diseases [28,29]. We suggest that humans with the different allele variants of the *ADRA1A* gene may have adaptive variability in vessel reactions during imitation of the diving reflex.

Further, the hormonal background may significantly modulate the reflective adrenergic and cholinergic reactions [30]. Thus, it is well known that testosterone elevates α_1A_-AR expression [31] and decreases adenylate cyclase expression [32], which in turn significantly increases the vessel tonus and arterial blood pressure in men. It has also been reported that estrogen increases endothelial β_1_- and β_2_-ARs expression, which stimulates NO-dependent vessel relaxation in women in response to adrenergic stimulation [33]. It has also been established that female sex hormones promote the vagus nerve-mediated reactions [34]. Thus, we can suggest that, in women, reflective vessel constriction is less manifested, while the cholinergic influence is more pronounced, when compared to men.

Within this study, we aimed to investigate the adaptive lung vessel reactions in response to imitation of the diving reflex and its variations in men and women with *Arg347Cys (rs1048101)* in the *ADRA1A* gene. These studies are significant, as they elucidate the mechanisms of lung edema and hypertonia associated with hypoxia.

## 2. Results

*Changes in the pulmonary arterial system in response to the diving reflex reveals inconsistency in the study group*. All subjects completed the protocol, had no special physical training, and had never practiced free diving. In accordance with instructions, subjects did not hyperventilate before face immersion. Table 1 presents the anthropometric data, where no significant sexual or body mass index (BMI) differences were found.

The average duration of apnea during the imitation of the diving reflex in the study group was 35.0 ± 14.1 s. In all subjects, the alveolar PO_2_ was significantly reduced, while the alveolar PCO_2_ was significantly increased in the exhaled air after breath holding with face immersion in water compared with the control level (*p* < 0.05). In ambient air, the results were as follows: PO_2_ = 158.0, PCO_2_ = 0.26 mm Hg; before apnea—PO_2_ = 122.0 ± 7.5 mm Hg, PCO_2_ = 38.3 ± 2.3 mm Hg; at end of the diving reflex imitation—PO_2_ = 97.1 ± 8.3 * mm Hg, PCO_2_ = 49.5 ± 6.9 * mm Hg.

**Figure 1:**
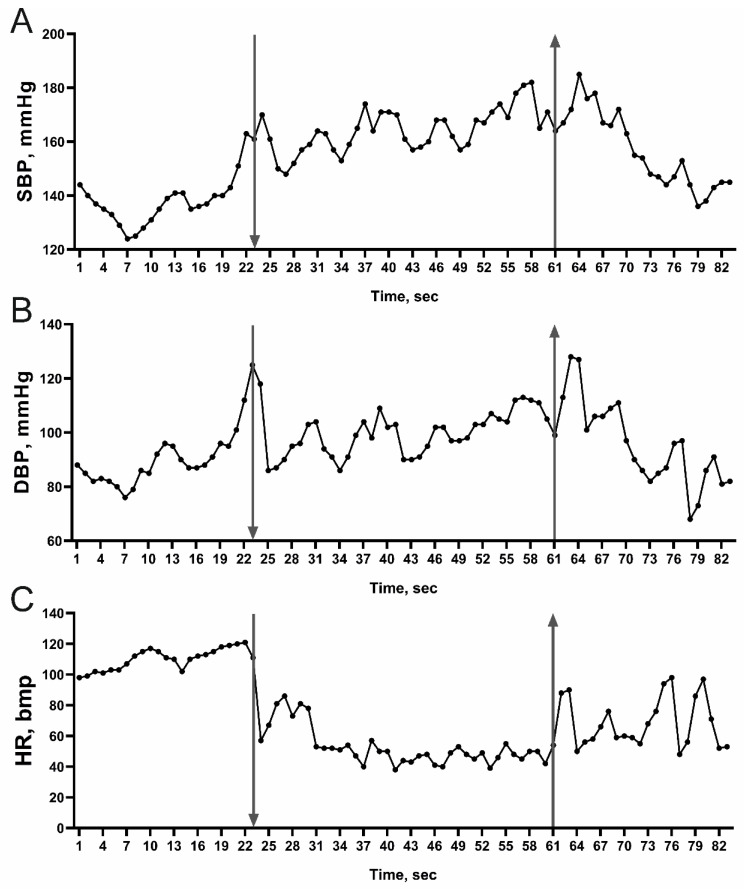
Representative dynamic of blood pressure and heart rate changes in the subject M during imitation of the diving reflex. Arrow pointing down indicates the beginning of face submersion; arrow pointing up indicates the end of face submersion. (**A**) Changes in systolic blood pressure (SBP) during the imitation of the diving reflex. (**B**) Changes in diastolic blood pressure (DBP) during the imitation of the diving reflex. (**C**) Changes in heart rate (HR) during the imitation of the diving reflex.

As expected, all of the subjects demonstrated the presence of bradycardia in response to face immersion (FIm) during the imitation of the diving reflex, which was associated with systolic (SBP) and diastolic (DBP) blood pressure increase (Figure 1 and Table 2). Analysis of the blood flow in men and women enrolled in the study showed a significant reduction in the pulse wave amplitude (PWA) in response to the imitation of the diving reflex (Table 2), which indirectly reflects a reduction in the peripheral blood flow velocity.

**Figure 2:**
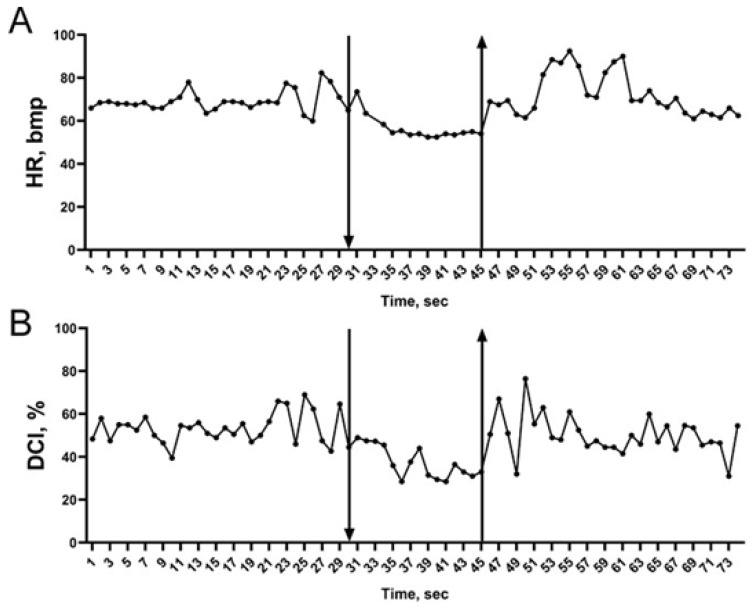
Representative dynamics of cardiac rhythm and pulmonary vascular dicrotic index changes in the subject M during imitation of the diving reflex. Arrow pointing down indicates the beginning of face submersion; arrow pointing up indicates the end of face submersion. (**A**) Changes in heart rate (HR) during imitation of the diving reflex. (**B**) Changes in dicrotic index (DCI) during imitation of the diving reflex.

Further, to evaluate lung blood flow, we used the following parameters: RI—rheographic index of the right pulmonary arterial system, which indirectly indicates the level of blood filling into the pulmonary artery during right ventricle blood output; DCI—dicrotic index, which is an indirect indication of the vascular tone in pulmonary artery large vessels; and DSI—diastolic index, which helps to characterize the ratio of venous drainage to arterial blood supply in the right lung [35]. While no changes were found in RI between the face immersion attempts, DCI and DSI were significantly lower during the face submersion at attempts #1 and #2 but not at attempt #3 (Table 2). Interestingly, 54% of the subjects demonstrated a stable reduction in DCI and DSI, while 41% of the subjects had an unstable reduction in DCI and DSI (i.e., at least one attempt at face submersion was not accompanied by DCI and DSI decrease), and only 5% of the subjects reacted with an increase in DCI and DSI in response to imitation of the diving reflex. Individual changes in DCI are shown in Figure 2.

Analysis of the latent period of bradycardia development (1–10 s), time of peripheral vasoconstriction development (1–3 s, based on the PWA reduction), time of DCI reduction (0.6 to 10 s), and time of changes in blood pressure (6.8 to 43.5 s) indicates the reflective nature of these changes and suggests that the pulmonary vascular tone changes irrelatively to changes in blood pressure during imitation of the diving reflex (Figure 1 and Figure 2).

Overall, reduction in DCI and DSI during face submersion suggests that the vascular tone in the pulmonary arterial system is diminished, which indirectly indicates improvements in lung perfusion with blood. However, the high variability in the individual reactions of the subjects to face submersion led us to suggest that sexual dimorphism may play a significant role.

*Men and women have differences in their cardiovascular manifestation in response to imitation of the diving*. Further analysis of the possible sex-related peculiarities in response to face submersion revealed significantly increased RI in women when compared to men (Table 3). Interestingly, this parameter remained higher before, during, and after imitation of the diving reflex. In contrast, parameters such as SO, PWA, and SBP were significantly higher in men before, during, and after imitation of the diving reflex, while a significant increase in the DCI and DSI parameters was found in men during face submersion only (Table 3). Overall, these data clearly demonstrate sexual dimorphism in the cardiovascular reactions in response to the diving reflex.

*The vascular reactions in the pulmonary system are associated with the ADRA1A gene polymorphism in men and women*. To validate our findings in the context of a possible involvement of the *ADRA1A* gene, we analyzed the *Arg347Cys (rs1048101)* genotype in our study cohort. We found a higher C/T presence in both men and women (Table 4).

Analysis of the rheographic parameters of the pulmonary arterial system revealed no correlation between differences in RI, DCI, and DSI and either C/T, C/C, or T/T genotypes in the study subjects. However, in women with C/T and T/T polymorphisms, RI was significantly higher before, during, and after imitation of the diving reflex when compared to men with C/T and T/T polymorphisms (Table 5). Interestingly, the C/C polymorphism did not contribute to the RI changes. These findings suggest that vascular blood filling of the pulmonary arterial system is higher in women than in men. Furthermore, in men with the C/T polymorphism, DCI and DSI were significantly higher when compared to women with C/T allele presence (Table 6 and Table 7).

**Figure 3:**
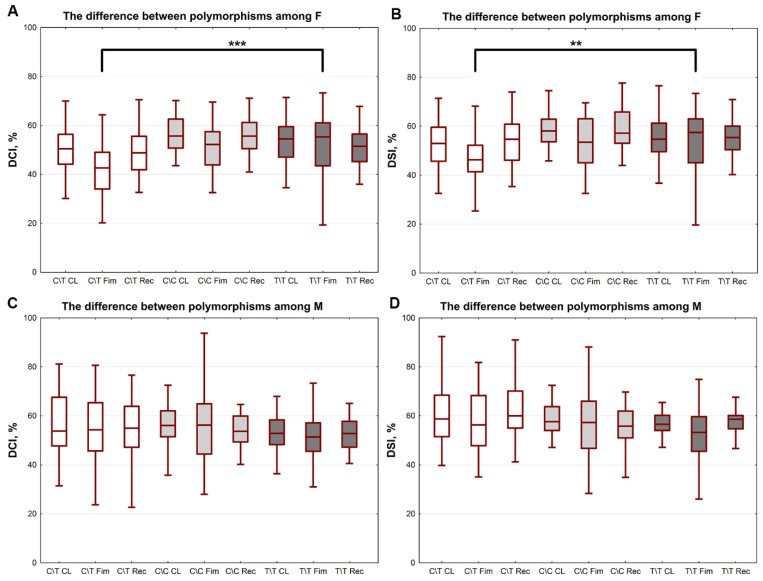
Dynamic of pulmonary blood flow in women with different *ADRA1A* gene polymorphisms. (**A**) Dicrotic index (DCI) and (**B**) diastolic index (DSI) in women with C/T, C/C, and T/T allele variants during imitation of diving reflex. (**C**) DCI and (**D**) DSI in men with C/T, C/C, and T/T allele variants during imitation of diving reflex. CL—control level, FIm—face immersion, Rec—recovery after face submersion. **—*p* < 0.01; ***—*p* < 0.001; *n* = 50.

Analysis of the variations in the circulation parameters inside the groups of women and men demonstrated no changes among men. However, the group of women with the C/T polymorphism had lower DCI and DSI parameters in response to the diving reflex imitation when compared to women with the T/T polymorphism (Figure 3).

## 3. Discussion

In this study, we investigated the adaptive reactions of the human body in response to the diving reflex as a hypoxia model. Human beings with no adaptation to diving were used in the study, which allowed us to characterize the adaptive reactions typical for *Homo sapiens* with no phenotypic adaptation. In the entire study cohort, we detected a significant decrease in heart rate and a significant increase in peripheral vessel tonus (based on data from the plethysmogram) and blood pressure, which is in accordance with data previously published by us and others [36,37]. Peripheral vasoconstriction is a universal adaptive reaction of any organism in response to hypoxia of any genesis which helps to redistribute blood flow to the most oxygen-unresistant organs, such as the brain and heart. Notably, in our previous study, we demonstrated for the first time significantly lower tonus of the pulmonary artery in response to the diving reflex [17], which was confirmed again in the present work. We suggest that this is another reflective reaction similar to the reflective bradycardia and peripheral vasoconstriction of the systemic circulation vessels and that the vessels’ dilation in the pulmonary circulation is a part of the diving reflex.

The reflective vessel dilation in the pulmonary circulation may have a dual explanation. First, it might be associated with the mechanism of interaction between the systemic and pulmonary circulations. The constant tonic influence of the sympathetic nervous system has an impact on the vessels of the systemic and pulmonary circulations. In response to the diving reflex, the baroreceptors of the carotid sinus become excited due to reflective vasoconstriction and increased blood pressure, which reflectively suppresses the vasomotor center’s activity and, consequently, the activity of the sympathetic fibers. In turn, this results in decreased vessel resistance of the pulmonary circulation and leads to an increased blood supply in the lungs and normalization of the blood pressure in the systemic circulation.

However, our data indicate that dilation of the lung vessels in response to the diving reflex occurs prior to the blood pressure increase, as the latent time of the blood pressure increase was 6.8–43.5 s, while the latent time of the lung vessel dilation was 0.6–10.1 s. Therefore, we suggest that lung vessel dilation may be associated not with a decrease in the sympathetic activity only [19] but with a reflective increase in the parasympathetic activity of the vagus nerve in response to the diving reflex, similar to the increased influence of the parasympathetic part of the autonomic system on the heart [38]. This occurs as a consequence of the increased afferent influences from the trigeminal nerve in response to the activation of the cold and tactile receptors of the face skin while submerged. At the same time, the reflective parasympathetic cholinergic activation results in decreased tonus of the lung vessels via the M3-cholinoreceptors located in the endothelium of the lung vessels [39]. The “cold factor”, which is in place during imitation of the diving reflex, increases the parasympathetic activity and may lead to an increase in the adrenergic influences, where β1- and β2-adrenoceptor activation will result in vessel dilation [21], but α1-adrenoceptor activation will result in vessel constriction [16]. It has been shown that the fast reflective sympathetic reaction of vessels occurs via α1A-adrenoceptors [40], suggesting that these receptors play a pivotal role in the regulation of the lung vessels’ tone.

In our present study, many of the parameters of the cardiovascular system had high variability, which might be explained by the difference in sex hormones. Indeed, the analysis of the same parameters between men and women showed much less variability and demonstrated a number of significant changes.

Thus, analysis of the cardiovascular parameters before and during imitation of the diving reflex revealed that men had higher levels of stroke volume and systolic blood pressure in association with higher levels of blood fulfillment in the peripheral vessels. However, women had higher levels of resistive vessel constriction during the imitation of the diving reflex, which might be explained by higher levels of sympathetic nervous system activity [41]. We also showed that women had higher lung fulfillment with blood, which might be linked to higher levels of peripheral vessel tonus and, consequently, blood redistribution to the lung. Interestingly, women had decreased tonus of the lung vessels in response to imitation of the diving reflex when compared to men.

Previously, it was demonstrated that testosterone increased the expression of the α1-adrenoceptors [42]. This may cause more pronounced vasoconstriction in men than in women. In addition, sex variations may be due to differences in the structure and function of the autonomic nervous system, which is affected by the sex hormones. These differences are mediated by the presence of XX or XY chromosomes, where the presence of the Y chromosome and testosterone production have a pivotal role in the formation of the “masculine type” of brain development and determine the differences in autonomic nervous system formation [43,44]. As a result, this affects autonomic system activity and the type of synaptic signal transduction [45]. At the same time, studies suggest an association between the female sex hormones and blockade of sympathetic nervous system activation by the vagus nerve [17,46,47,48,49,50,51].

Notably, during the last few years, sex hormone variations have been associated with the ovarian cycle in women, which in turn may result in higher variability of the physiological parameters. As a consequence, studies performed using men only resulted in a reduction in the physiological variability. However, a recent study has shown higher variability in physiological parameters in men than in women [52]. In agreement with this study, our data showed that women had lower pulmonary artery tonus during imitation of the diving reflex compared to men, which is rather associated with the Arg347Cys *ADRA1A* gene polymorphism than with the ovarian cycle. However, the relationship between different stages of the ovarian cycle and changes in vascular tone in women carrying different SNP alleles needs to be investigated further.

In addition, our study demonstrated no differences in the physiological parameters tested when no sex differences were taken into account, which was in accordance with the previous study that revealed an absence of any differences in vitro in the Arg347Cys receptor compared to the wild type [27]. However, sex difference analysis revealed higher lung vessel dilation in 95.3% of women with the CT allele, while only 64% of men demonstrated vasodilation in the lungs. We suggest that this might be explained by the presence of estrogen, which enhances the reactions via the vagus nerve and blocks sympathetic activation [50]. Further, we demonstrated the presence of significant changes between the women with CT (p.Arg347Cys *ADRA1A*) and TT (*pCys347Cys ADRA1A*) alleles, where women with the CT allele had higher levels of lung vessel tonus reduction. Notably, four out of seven women with the TT allele had increased tonus of the lung vessels. Interestingly, 82% of men and women with the CC allele (*pArg347Arg ADRA1A*) had decreased tonus of the lung vessels. Thus, we can suggest that women with the *Cys347Cys* mutation have less sensitivity to the effect of the sex hormones. At the same time, data on the Chinese population have demonstrated an association between the Cys347 allele and sensitivity to nifedipine, a drug used against hypertension [53]. Another study from Brazil (*n* = 1500) found that the allele *Cys rs1048101* was associated with higher levels of blood pressure in physically active young participants but not in sedentary ones [54]; this may help them to adapt faster to changes in the environment. However, our data from participants with no adaptation to diving suggest that women with the allele *Arg rs1048101* have better mechanisms of adaptation to diving when compared to *Cys rs1048101*. However, these data require further investigation and theoretical understanding using a larger cohort. At the same time, these are unique data, as they were collected from humans with no adaptation to diving and may represent the native physiological reactions to the diving reflex as a result of the human phylogenetic process. Historically, it has been noted that women have a priority position in diving irrespective of their nationality [55], which is very possibly associated with the peculiarities of the autonomic nervous system reactions with regards to the sex hormone status in women. Further, hormonal status is the key factor which causes the polymorphic genome organization that helps to identify women with the greatest diving adaptation capabilities.

In conclusion, the mechanisms of the diving reflex, which protects the human body from extreme environmental factors such as hypoxia, remain far away from full understanding. However, our data showed that lung fulfillment with blood started from the reflective vasodilation of the lung vessels and that women had higher levels of this dilation. Women with the Arg347 rs1048101 allele had the greatest adaptive capabilities. However, this observation requires further investigation in professional divers. This is important, as these studies may shed light on the mechanisms of the diving reflex in humans and on how we could use it to improve overall health and to control the autonomic nervous system in order to prevent the formation of lung edema in response to hyper- and hypobaria.

## 4. Materials and Methods

*Subject recruitment and data collection*. One hundred and two healthy volunteers (50 women and 52 men) without special physical training were included in the study. All of the subjects (students at the Saint-Petersburg State University, Russia) were informed about the study purposes, objectives, and methods and gave their voluntary consent by signing the Informed Consent Form. All of the subjects participated in the study voluntarily and had no direct benefit from the test (financial rewards, educational requirements, or credits). Information about the general results of the research or personal data (such as genetic variants) was provided for individuals who were interested. At the time of the experiment, none of the volunteers had arteriosclerosis or diabetes or were taking any drugs. The subjects were asked to skip smoking and coffee at least two hours prior to the study. A short list of the groups’ characteristics is presented in Table 1. The study was approved by the Saint-Petersburg State University Ethics Review Committee for human studies (No. 40 from 7 March 2022).

*Experimental model of the diving reflex in humans*. Activation of the diving reflex was performed by face submersion in cold water under laboratory conditions. As it is well known [5] that a 10 °C gradient between air temperature and water temperature is optimal for the manifestation of the diving reflex, in our experiments, the water temperature was 14.3 ± 3.0 °C, and the air temperature was 21.9 ± 1.5 °C. Prior to the start of the experiment, all subjects stayed in the laboratory for at least 40 min and were adapted to the local temperature. The procedure was performed on a subject lying in a ventricumbent position on a couch with arms along the body (as published previously in [22]). During an experimental procedure, all subjects kept their hands at the heart line, did not change this position, and did not move their fingers equipped with a finger sensor. Three face submersions on a normal exhalation were performed in cold water. The duration of the first submersion was limited to the first feeling of discomfort. After the first face submersion, which we considered as an orienting one, a full recovery of cardiovascular parameters was conducted within 10 min. The pause between submersions was 2–3 min.

*Measurements of the physiological parameters*. Before the experimental procedure, an electrocardiogram (ECG) was recorded in I standard lead and checked for abnormalities. During the whole experiment (rest, imitation of the diving reflex, and recovery), ECG, blood pressure (BP), and central blood flow were constantly recorded. The rheogram was recorded by Tishchenko’s integrated body rheography method (Tishchenko’s impedance cardiographic method of total systemic blood flow assessment) [56] using RGPA-6/12 “Rean-Poly” (Medicom-MTD, Taganrog, Russia). A relative index of skin capillary blood flow in the left index finger was recorded by a photoplethysmogram (PPG). The pulmonary artery rheogram was recorded by the impedance method for the investigation of right pulmonary artery blood flow (RPABF) [35,57,58], and the following physiological parameters were used for the evaluation: stroke output (SO, mL), rheographic index (RI, Ohm; reflects the lung tissue blood filling), dicrotic index (DCI, %; reflects the vascular tone of resistant vessels of the pulmonary artery system), and diastolic index (DSI, %; reflects the ratio of venous drainage to arterial blood supply).

Pulse wave amplitude (PWA, pm) [59] was calculated based on the photopletismogram records using “Rean-Poly” software (Elite version). It has previously been reported that PWA indirectly reflects vascular perfusion of the distal phalanx of the hand and essentially depends on the sympathetic influences of the autonomic nervous system [60]. Heart rate (HR) and systolic and diastolic BP were registered by the oscilloscopic method (AND UA-797, Tokyo, Japan). To determine the BP latency period, a noninvasive method with a Finometer (FMS, Purmerend, The Netherlands) was used.

Respiratory flow and volume, as well as expiratory O_2_ and CO_2_ fractions (pO_2_ and pCO_2_) for the determination of alveolar gas exchanges, were recorded with a microprocessor analyzer (MF01, Research and Production Center for Environment and Health—CEZ, Russia).

*DNA isolation and PCR*. Blood samples for DNA isolation were collected from a finger using biomaterial sampling and storage BIOCHRAN cards. DNA samples from the blood of all patients were isolated by phenol chloroform extraction as described previously [61], and concentration was determined using Qubit software (Invitrogen) with Qubit DNA HS Assay Kits according to the manufacturer’s instructions. ADRA1A gene polymorphisms (p.Arg347Cys; rs1048101) were tested by the polymerase chain reaction-restriction fragment length polymorphism (PCR-RFLP) method. Nucleic acid sequences of the gene fragments of interest were obtained from the Nucleotide Network Base (NCBI, Bethesda, MD, USA). The primers required for their amplification were selected using the Oligo 6 program (USA), and its specificity was tested using Nucleotide-nucleotide BLAST (NCBI, Bethesda, MD, USA). A polymorphism (rs1048101) in the ADRA1A gene was analyzed using the following primers: 5′–CATCATATACCCATGCTCCAGC–3′ (forward) and 5′–CATCCGTCTTGGAGATCCTGTA–3′ (reverse).

The 25 µL PCR mix included deionized water, 1.5 mM MgCl_2_, 0.5 mM of each dNTP (Silex, Russia), 0.5 U of *Taq* polymerase (Silex, Russia), a mix of forward and reverse gene-specific primers at 0.2 pM each, 1 µL of DNA template, and a PCR buffer (6.7 mM Tris·HCl, pH 8.6, 16.6 mM NH2SO4, and 0.001% Triton X-100). After the initial denaturation of the sample at 95 °C for 5 min, we carried out 37 cycles as follows: 95 °C for 30 s; 60 °C for 30 s; and 72 °C for 1 min. A final incubation for 5 min at 72 °C was added to complete the DNA synthesis. The PCR product c.1039C > T ADRA1A (rs1048101) was digested with Pst I endonuclease at 37 °C overnight in 15 μL reaction mix containing 5 μL of amplicon, 8 μL of ampouled water, 1.5 μL of 10x buffer (as recommended by the manufacturer), and 0.5 μL of restriction enzyme. Restriction products were separated by means of electrophoresis (180 volts for the first 15 min and 380 volts afterwards) and visualized after ethidium bromide staining in transmitted UV light.

*Statistical analysis*. Statistical analysis was performed using the statistical package GraphPad Prism 8 for Windows 10. The values were expressed as means ± SE.

The normality of distribution was tested using the chi-square test. The significance of differences in normally distributed samples was evaluated using Student’s t-test and one-way ANOVA.

To assess the significance between samples with deviations from a normal distribution and between small samples (comparison of groups with different combinations of SNP Arg347 ADRA1A (rs1048101) alleles), non-parametrical Mann–Whitney and Kruskal–Wallis rank analysis of variance tests were used.

Moreover, *p*-values < 0.05 were considered to be statistically significant.

## Figures and Tables

**Table 1:** Anthropometric parameters of the studied group.

Parameter	Men (*n* = 52)	Women (*n* = 50)
**Weight, kg**	73.4 ± 10.7	58.6 ± 8.5
**Height, cm**	178.9 ± 6.2	165.7 ± 6.2
**BMI**	22.8 ± 2.5	21.4 ± 3.1
**Age, yr**	24.2± 4.7	21.9 ± 2.9

Note: BMI—body mass index.

**Table 2:** Blood flow parameters from the subjects enrolled in the study before, during, and after the diving reflex imitation.

Parameter	Attempt #1			Attempt #2			Attempt #3		
	CL	FIm	Rec	CL	FIm	Rec	CL	FIm	Rec
**HR, bmp**	74.3 ± 1.2	65.3 ± 1.3 ***	68.4 ± 1.2	72.9 ± 1.1	63.5 ± 1.4 ***	67.9 ± 1.1	73.3 ± 1.2	63.5 ± 1.2 ***	67.8 ± 1.1
**SO, mL**	91.9 ± 2.1	93.9 ± 2.4	95.8 ± 2.2	93.1 ± 2.2	95.2 ± 2.33	96.4 ± 2.1	95.1 ± 2.1	96.4 ± 2.2	97.0 ± 2.1
**PWA, pm**	0.92 ± 0.09	0.39 ± 0.03 ***	1.09 ± 0.10	0.93 ± 0.09	0.41 ± 0.04 ***	1.06 ± 0.10	0.80 ± 0.08	0.36 ± 0.03 ***	0.98 ± 0.09
**RI, Ohm**	0.30 ± 0.014	0.30 ± 0.018	0.29 ± 0.009	0.29 ± 0.009	0.28 ± 0.008	0.30 ± 0.009	0.29 ± 0.008	0.27 ± 0.008	0.28 ± 0.008
**DCI, %**	56.16 ± 1.03	51.41 ± 1.4 ***	53.68 ± 0.96	55.31 ± 0.9	50.3 ± 1.4 **	54.21 ± 0.94	54.74 ± 0.94	52.32 ± 1.4	53.27 ± 0.99
**DSI, %**	58.09 ± 0.97	52.26 ± 1.4 ***	58.21 ± 0.9	57.92 ± 0.9	52.87 ± 1.4 **	59.16 ± 0.94	57.57 ± 0.93	54.88 ± 1.4	58.50 ± 1.0
**SBP, mmHg**	115.1 ± 0.99	129.4 ± 1.63 ***	125.9 ± 1.08	116.3 ± 0.96	129.5 ± 1.61 ***	117.3 ± 0.92	117.9 ± 0.89	127.9 ± 1.51 ***	116.4 ± 1.04
**DBP, mmHg**	69.41 ± 0.63	83.64 ± 1.09 ***	69.84 ± 0.63	68.7 ± 0.92	82.89 ± 1.06 ***	70.63 ± 0.60	70.8 ± 0.68	83.32 ± 1.08 ***	70.51 ± 0.67

Note: HR—heart rate; SO—stroke output; PWA—pulse wave amplitude; RI—rheographic index of the right pulmonary arterial system; DCI—dicrotic index; DSI—diastolic index; SBP—systolic blood pressure; CL—control level; FIm—face immersion; Rec—recovery after face submersion. **—*p* < 0.01; ***—*p* < 0.001 is statistically significant between CL and FIm; *n* = 102.

**Table 3:** Sex-related differences in the blood flow parameters before, during, and after diving reflex imitation.

Parameter	Women (*n* = 50)			Men (*n* = 52)		
	CL	FIm	Rec	CL	FIm	Rec
**HR, bmp**	73.75 ± 0.98	63.39 ± 1.13	68.22 ± 0.88	74.23 ± 1.29	66.02 ± 1.21	69.51 ± 1.30
**SO, mL**	79.06 ± 1.15	79.69 ± 1.29	82.20 ± 1.05	107.10 ± 1.45 ***	109.70 ± 1.57 ***	109.80 ± 1.53 ***
**PWA, pm**	0.58 ± 0.05	0.3 ± 0.03	0.7 ± 0.06	1.13 ± 0.09 ***	0.45 ± 0.03 **	1.28 ± 0.09 ***
**RI, Ohm**	0.31 ± 0.01 ***	0.32 ± 0.01 ***	0.31 ± 0.01 ***	0.26 ± 0.01	0.24 ± 0.01	0.27 ± 0.01
**DCI, %**	52.99 ± 0.78	47.70 ± 1.05	51.27 ± 0.76	55.03 ± 0.82	53.52 ± 1.23 ***	53.62 ± 0.85
**DSI, %**	55.34 ± 0.78	50.54 ± 1.06	55.77 ± 0.75	58.06 ± 0.7673	56.32 ± 1.22 ***	58.83 ± 0.81
**SBP, mmHg**	110.2 ± 1.4	125.2 ± 2.1	112.0 ± 1.3	119.5 ± 1.2 **	133.7 ± 2.6 **	119.0 ± 1.1 *
**DBP, mmHg**	68.57 ± 0.96	83.26 ± 1.48	69.50 ± 0.97	70.02 ± 0.85	83.70 ± 1.71	70.02 ± 0.86

Note: HR—heart rate; SO—stroke output; PWA—pulse wave amplitude; RI—rheographic index of the right pulmonary arterial system; DCI—dicrotic index; DSI—diastolic index; SBP—systolic blood pressure; CL—control level; FIm—face immersion; Rec—recovery after face submersion. *—*p* < 0.05; **—*p* < 0.01; ***—*p* < 0.001 is statistically significant between women and men in each state during the imitation of the diving reflex.

**Table 4:** The genotype distribution of *ADRA1A p.Arg347Cys* (rs1048101) in the study population.

*ADRA1A* (rs1048101)	C/T	T/T	C/C
**Total (*n* = 102)**	47	23	32
**Women (*n* = 50)**	21	12	17
**Men (*n* = 52)**	26	11	15

**Table 5:** Difference in rheographic index (RI, Ohm) between men and women with matched *ADRA1A p.Arg347Cys* (rs1048101) genotypes.

Genotype	Women (*n* = 50)			Men (*n* = 52)		
	CL	FIm	Rec	CL	FIm	Rec
**C/T**	0.315 ± 0.012 **	0.313 ± 0.013 ***	0.308 ± 0.011 *	0.257 ± 0.008	0.246 ± 0.008	0.264 ± 0.009
**T/T**	0.307 ± 0.013 *	0.3438 ± 0.03 ***	0.3101 ± 0.01 *	0.2536 ± 0.014	0.2211 ± 0.009	0.2598 ± 0.01
**C/C**	0.3484 ± 0.038	0.3245 ± 0.016	0.3296 ± 0.02	0.2876 ± 0.01	0.2631 ± 0.01	0.2835 ± 0.01

Note: CL—control level; FIm—face immersion; Rec—recovery after face submersion. C/T—F, *n* = 21; M, *n* = 26; C/C—F, *n* = 12; M, *n* = 11; T/T—F, *n* = 17; M, *n* = 15. *—*p* < 0.05; **—*p* < 0.01; ***—*p* < 0.001 is statistically significant between women and men with the same genotype in the same state during imitation of the diving reflex (CL F—CL M, FIm F—FIm M, Rec F—Rec M).

**Table 6:** Difference in dicrotic index (DCI, %) between men and women with matched *ADRA1A p.Arg347Cys* (rs1048101) genotypes.

Genotype	Women (*n* = 50)			Men (*n* = 52)		
	CL	FIm	Rec	CL	FIm	Rec
**C/T**	50.49 ± 1.13	43.36 ± 1.58	49.13 ± 1.11	56.28 ± 1.29 *	55.00 ± 1.67 ***	54.98 ± 1.37 *
**T/T**	54.3 ± 1.21	51.8 ± 1.79	51.97 ± 1.24	51.61 ± 1.21	48.78 ± 2.10	51.44 ± 1.01
**C/C**	55.93 ± 1.89	49.76 ± 1.88	54.47 ± 1.69	56.23 ± 1.47	55.78 ± 2.94	53.04 ± 1.70

Note: CL—control level; FIm—face immersion; Rec—recovery after face submersion. C/T—F, *n* = 21; M, *n* = 26; C/C—F, *n* = 12; M, *n* = 11; T/T—F, *n* = 17; M, *n* = 15. *—*p* < 0.05; ***—*p* < 0.001 is statistically significant between women and men with the same genotype in the same state during imitation of the diving reflex (CL F—CL M, FIm F—FIm M, Rec F—Rec M).

**Table 7:** Difference in diastolic index (DSI, %) between men and women with matched *ADRA1A p.Arg347Cys* (rs1048101) genotypes.

Genotype	Women (*n* = 50)			Men (*n* = 52)		
	CL	FIm	Rec	CL	FIm	Rec
**C/T**	53.71 ± 1.22	47.39 ± 1.62	54.6 ± 1.23	59.58 ± 1.25 *	58.1 ± 1.61 ***	61.13 ± 1.24 *
**T/T**	55.89 ± 1.13	53.91 ± 1.79	55.78 ± 1.06	54.98 ± 0.97	51.87 ± 2.08	57.4 ± 1.01
**C/C**	57.76 ± 1.83	51.4 ± 2.00	58.14 ± 1.69	58.21 ± 1.36	57.52 ± 3.11	55.09 ± 1.68

Note: CL—control level; FIm—face immersion; Rec—recovery after face submersion. C/T—F, *n* = 21; M, *n* = 26; C/C—F, *n* = 12; M, *n* = 11; T/T—F, *n* = 17; M, *n* = 15. *—*p* < 0.05; ***—*p* < 0.001 is statistically significant between women and men with the same genotype in the same state during imitation of the diving reflex (CL F—CL M, FIm F—FIm M, Rec F—Rec M).

## Data Availability

The data generated in the present study are available from the corresponding author upon reasonable request.
